# Optimal reduction and conversion of range-difference measurements for positioning

**DOI:** 10.1371/journal.pone.0273617

**Published:** 2022-08-29

**Authors:** M. Hou

**Affiliations:** Department of Engineering, University of Hull, Hull, United Kingdom; University of Bradford, UNITED KINGDOM

## Abstract

For positioning an object with *m* references, there are *m*−1 linearly independent range differences and measuring them is essential. However, none of *m*(*m*−1) possible range differences should be considered redundant unless their measurements are free of noise and locations of the references are exactly known. From all available range-difference measurements, *m* range measurements are obtained for positioning based on the least squares principle. The problem formulation treats missing and weighted range-difference measurements simultaneously. The exact relationships among several formulations of least squares positioning are established. A numerical example illustrates the results.

## Introduction

Positioning an energy-emitting or reflecting object is an intensively studied topic due to its importance in wide applications [1]. Often it is based on the principle of time differences of arrival (TDOA), which means use of indirect measurements of range differences between the object at an unknown location and references at known locations. Positioning with TDOA measurements is different but closely related to that using time of arrival (TOA) measurements with or without a bias. With all possible range measurements between each object pair, positioning multiple objects is possible [2].

Positioning an *n*D object with *m* references requires *m* > *n* and up to *m*(*m*−1) range differences can be formed, but only *m*−1 of them are linearly independent. Due to noise effects, measurements of all available range differences should be used for positioning in applications. This work shows how to combine all available TODA measurements to form *m* TOA measurements for least squares positioning. Examinations of least squares criteria of several types of TOA and TDOA measurement equations establish equivalent and other exact relationships among these positioning formulations. The cases of positioning with missing and weighted TDOA measurements are treated inclusively in this work.

Investigation on the underlying problem is important from both theoretical and practical viewpoints. This kind of study answers the question of whether or not different TODA and TOA formulations are equivalent for positioning, and provides simplified equations for algorithm development and implementation in applications.

## Related work

For positioning an *n*D object with *m* known references, most methods have used *m* TOA equations or *m*−1 TODA equations for positioning, and normally the minimum number of references *m* = *n* + 1 is assumed. In the majority of TDOA methods, measurements of the remaining possible (*m*−1)^2^ range differences are unused or assumed to be unavailable.

In an early study [3], a large number of TDOA measurements with the minimum number of references were combined to form TODA triads for improvement on positioning. Optimality of the combination was not addressed nevertheless.

The problem of TDOA denoising [4] is to find a range-measurement vector for generation of an ideally structured TDOA measurement matrix closest to the original noise-corrupted same matrix by least squares. This problem is related but not equivalent to the TDOA positioning problem directly addressed in the current study.

The problem formulation in the current study avoids the assumptions on skew symmetry of the noise-corrupted TDOA measurement matrix, and on Gaussian distribution of the noise [4, 5]. This allows a more general coverage of noise conditions and consideration of up to *m*(*m*−1) TDOA measurements rather than half of them. Normally missing and weighted TDOA measurements are treated separately, for instance, in [4, 6], but simultaneously in the current study.

The focus of this work is on optimal conversion of range-difference equations rather than solving them. This is because, assuming exact solvability, closed-form solutions are known for positioning with *m* biased TOA measurements [7–12], and with *m* − 1 TDOA measurements [13–15]. As well known, *m* TOA measurements can be trivially converted to *m* − 1 TDOA measurements, although optimality of such a conversion is unclear. In real applications, closed-form solutions offer fine approximations, and can also be used to initiate an iterative algorithm for improving the solutions. These methods can be applied to the *m* TOA or further *m* − 1 TDOA measurement equations converted from possible *m*(*m* − 1) range-difference equations studied in the current work.

## Notations

All considered quantities are real numbers. Scalars are lowercase letters, (column) vectors and matrices are boldfaced lowercase and uppercase letters respectively. Set {*a*_*i*_} contains elements with a known number, and they can form a vector **a** = [*a*_*i*_]. The vector of ones is denoted by **e**. **A** = [*a*_*i,j*_] is a matrix of a known size with *a*_*ij*_ being its element in the *i*th row and *j*th column. Diagonal matrix **D**_**a**_ has elements of **a** on its diagonal, and **D**_**e**_ is the identity matrix **I**. **A′**, tr**A**, rank**A** and **A**^+^ are the transpose, trace, rank and Moore-Penrose inverse of **A** respectively. The norm of **a** is $\left|a\right|=\sqrt{a^{\prime }a}$, and that of **A** is $\left|A\right|=\sqrt{\text{tr}(A^{\prime }A)}$. Denoted by **A** ∘ **B** is the entry-wise multiplication, namely Hadamard product of the two matrices of the same size. $A={\left(A^{\frac{1}{2}}\right)}^{\prime }A^{\frac{1}{2}}$ is a positive semi-definite matrix decomposed by its square root (matrix). **A** = **UΣV**′ is the singular value decomposition of **A** with **U′U** = **I**, **V′V** = **I**, and diagonal matrix **Σ** consisting of (non-negative) singular values of **A**. Denoted by arg min_x_ *f*(**x**) is the argument of the minimum of a scalar function, namely **x** minimizing *f*(**x**). Denoted by $n_{i}∼N\left(\bar{n},\sigma_{n}^{2}\right)$ is a random variable *n*_*i*_ satisfying the Gaussian distribution with mean $\bar{n}$ and variance $\sigma_{n}^{2}$. Similarly, $n∼N(\bar{n},\Sigma_{n})$ stands for a Gaussian random vector with mean $\bar{n}$ and variance matrix Σ_*n*_.

## Problem formulation

Denote the matrices of range differences and their measurements by **R** = [*r*_*ij*_] and **T** = [*τ*_*ij*_], respectively. On the TDOA principle, noisy measurements {*τ*_*ij*_} of range differences {*r*_*ij*_} are described by scalar equations

$$\begin{array}{c} \tau_{ij}=r_{ij}+n_{ij},\;i,j=1,2,\cdots ,m;\;\;i\neq j, \end{array}$$ (1)

where *n*_*ij*_ is a random variable with zero mean, *m* the number of references, and *r*_*ij*_ = *r*_*i*_ − *r*_*j*_ the difference of ranges *r*_k_ = |**p** − **p**_*k*_| for *k* = *i*, *j*, from an unknown object **p** to known references **p**_*i*_ and **p**_*j*_. If **p** is of dimension *n*, *m* > *n* is required. To have a unique **p** in the noise-free case, {**p**_*i*_} are assumed to be non-coplanar, namely they are not located in an (*n* − 1)D linear subspace.

Likely not all *m*(*m* − 1) measurements in (1) are available in applications even under the assumption that, without loss of generality, all *m* references have been used in generation of the measurements. Available measurements may also be weighted according to a priori knowledge of noise statistics. To consider cases of missing and weighted measurements simultaneously, define a masking matrix as

$$\begin{array}{c} E=\left[e_{ij}\right],\;e_{ij}=\{\begin{array}{cc} w_{ij}, & \tau_{ij}-\text{available,}\\ 0, & \tau_{ij}-\text{missing,} \end{array} \end{array}$$ (2)

where weight *w*_*ij*_ > 0, and in the case of non-weighting *w*_*ij*_ = 1.

Weights {*w*_*ij*_} in (2) could be chosen as the components of the inverse variance matrix of noise {*n*_*ij*_} in (1). This resembles the treatment of measurement noise in the Kalman filtering. However, the problem considered in this study is not the problem of tracking a moving target because, if any, dynamics of object **p** is not considered in the current study. Hence, positioning an object based on the equivalent range equations is generally not the minimum variance estimation intended with a Kalman filter.

Based on all available range-difference measurements and possibly also with weighting, positioning an object is to find a least squares solution of **p** to the matrix equation

$$\begin{array}{c} E\circ T=E\circ R. \end{array}$$ (3)

The objective of this study is to convert (3) which may have up to *m*(*m* − 1) range-difference equations to *m* range equations. The conversion is optimal in the sense of least squares.

In the case where no range-difference measurement is weighted or missing, the scalar equations in (1) are identical to the matrix equation in (3) except that the noise terms in the former are set to be zero in the latter. In general, (3) is a compact notation of (1) by setting the unknown noise to be zero but with simultaneous consideration of weighted and missing measurements. Clearly, (3) does not normally have an exact solution for **p**, and hence an estimation of **p** is sought in respect to least squares of (3).

## Linear dependence of {*r*_*ij*_} and properties of E

Range differences {*r*_*ij*_} are clearly related to each other, and linear independence of a subset of them is defined conventionally.

**Definition 1** $\left\{r_{i_{l}j_{l}}\right\}$ *for* *i*_*l*_, *j*_*l*_ ∈ {1, 2, …, *m*} *and* *l* = 1, 2, …, *k* *with an arbitrary integer* *k* > 0, *are said to be linearly independent from each other if* $\sum_{l=1}^{k}\alpha_{l}r_{i_{l}j_{l}}=0$ *implies coefficient* *α*_*l*_ = 0 *for all* *l*.

Range difference *r*_*ij*_ can be expressed as a linear combination of any *m* − 1 linearly independent elements in {*r*_*ij*_} for *i*, *j* = 1, 2, …, *m*. It is easy to verify that among others, {*r*_*i* + 1,1_}, {*r*_1,*i* + 1_} or {*r*_*i* + 1, *i*_} for *i* = 1, 2, …, *m* − 1, consists of *m* − 1 linearly independent elements. Clearly, in general, noise-corrupted range-difference measurements {*τ*_*ij*_} are linearly independent from each other.

Masking matrix **E** is non-negative, namely none of its components is negative. Also, **E** ≠ **0** because at least two range-difference measurements are available under the necessary condition *m* > *n* for unique positioning of an *n*D object with *m* references. If every reference has been used in the generation of TDOA measurements, for all *i*, the *i*th row and column of **E** cannot be simultaneously zero. This amounts to, for at least one *j*,

$$\begin{array}{c} e_{ij}+e_{ji}\neq 0,\;i=1,2,\cdots ,m. \end{array}$$ (4)

The case *e*_*ij*_ + *e*_*ji*_ = 0 for a particular *i* and all *j*, corresponds to non-use of the *i*th reference, which can be handled by dropping **p**_*i*_ and reducing number *m* by one in (1). As implied in (4), measurements of *m* − 1 linearly independent range differences are automatically available in (3).

## Pseudo range-measurement vector *τ* and properties of companion matrix $\bar{E}$

Define a companion matrix of **E** as

$$\begin{array}{c} \bar{E}=\left(E^{\prime }E+EE^{\prime }\right)\circ I-E\circ E-{\left(E\circ E\right)}^{\prime }, \end{array}$$ (5)

and a pseudo range measurement vector as

$$\begin{array}{c} \tau ={\bar{E}}^{+}\bar{\tau },\;\bar{\tau }=(E\circ E\circ T-{\left(E\circ E\circ T\right)}^{\prime })e, \end{array}$$ (6)

where ${\bar{E}}^{+}$ is the Moore-Penrose inverse of $\bar{E}$, and **e** the vector of ones.

In the special case where all *m*(*m* − 1) measurements in (1) are available and no weighting is applied to them, it is ready to obtain the simplifications **E** = **ee**′ − **I**, $\bar{E}=2m(I-\frac{1}{m}ee^{\prime })$ and $\tau =\frac{1}{2m}(T-T^{\prime })e$.

Companion matrix $\bar{E}$ is obviously symmetric, and $\bar{E}\neq 0$ due to **E** ≠ **0**. To explore its properties, some basic definitions related to matrix irreducibility are needed. These properties are important for reduction and conversion of the weighted range difference matrix equation in (3).

**Definition 2** *(Definition 6.2.25 [16]) Square matrix* **A** = [*a*_*i,j*_] *is said to be* irreducibly diagonally dominant *if*

- *it is* irreducible, *namely it is not similar to a block upper triangular matrix by permutation*.
- *it is* diagonally dominant, *namely* |*a*_*ii*_| ≥ ∑_*i*≠*j*_|*a*_*ij*_| *for all i*;
- *there is an i such that* |*a*_*ii*_| > ∑_*i*≠*j*_|*a*_*ij*_|.

**Theorem 1**

-
$\bar{E}$ *is diagonally dominant*;
-
$\bar{E}$ *is positive semi-definite*;
-
$|E\circ (xe^{\prime }-ex^{\prime })|=\left|{\bar{E}}^{\frac{1}{2}}x\right|$ *for arbitrary* x ;
-
$\bar{E}$ *is irreducible*;
-
$\text{rank}[\;\bar{E},\;\bar{\tau }\;]=\text{rank}\;\bar{E}=m-1$ .

## Equivalence and optimality of range and range-difference equations

In terms of an arbitrary vector **x** of dimension *m*, define a matrix as

$$\begin{array}{c} T_{x}=xe^{\prime }-ex^{\prime } \end{array}$$ (7)

which has the same structure as **R**, in fact **R** = **T**_**r**_ with range **r** = [*r*_*i*_]. For least squares positioning, exact relationships among (3) and the following three equations

$$\begin{array}{c} {\bar{E}}^{\frac{1}{2}}\tau ={\bar{E}}^{\frac{1}{2}}r,\;T_{\tau }=R,\;\tau =r+\bar{r}e \end{array}$$ (8)

with $\bar{r}=e^{\prime }\left(\tau -r\right)/m$, are shown in the next theorem.

**Theorem 2** *There are two least squares positioning equivalences:*

$$\begin{array}{c} \text{arg}\;\underset{p}{\text{min}}|E\circ (T-R){|}^{2}=\text{arg}\;\underset{p}{\text{min}}{\left|{\bar{E}}^{\frac{1}{2}}\left(\tau -r\right)\right|}^{2}; \end{array}$$ (9)

$$\begin{array}{c} \text{arg}\;\underset{p}{\text{min}}|T_{\tau }{-R|}^{2}=\text{arg}\;\underset{p}{\text{min}}{\left|\tau -r-\bar{r}e\right|}^{2}. \end{array}$$ (10)

*For arbitrary* **p**, *r*, *and* **E**, *the following relations hold*

$$\begin{array}{c} |{\bar{E}}^{\frac{1}{2}}\left(\tau -r\right)|/|{\bar{E}}^{\frac{1}{2}}|\leq |\tau -r-\bar{r}e|\leq |\tau -r-re|\leq |\tau -r|. \end{array}$$ (11)

The significance of Theorem 2 lies in establishment of the equivalence of matrix Eq (3) and that in (8) to the vector equations in (8) respectively through (9) and (10) for least squares positioning. Although further equivalence between (9) and (10) cannot be established, (11) implies that if a **p** diminishes $|\tau -r-\bar{r}e|$ considerably, it is a superb approximation of (9), and confirms the supremacy of $\tau =r+\bar{r}e$ over τ=r+re and τ=r for determination of **p** by the least squares principle.

**Corollary 1** *The denoising problem has the general solution*

$$\begin{array}{c} \text{arg}\underset{x}{\text{min}}{\left|E\circ (T-T_{x})\right|}^{2}=\tau -re \end{array}$$ (12)

*with an arbitrary r*.

The result presented in Corollary 1 was first obtained in [4], and now given without imposing any particular assumptions on **T** in (1) and **E** in (4). Theorem 2 and Corollary 1 indicate exactly the relationship between the positioning and denoising problems. Basically, for positioning $p=\text{arg}\;{\text{min}}_{p}{\left|E\circ (T-T_{r})\right|}^{2}$, while for denoising, $x=\text{arg}\;{\text{min}}_{x}{\left|E\circ (T-T_{x})\right|}^{2}$. In general, $|E\circ (T-T_{r}){|}^{2}\neq {\left|E\circ (T-T_{\tau -re})\right|}^{2}$, and the equality holds if **p** satisfies τ=r+re for some *r*. Note that normally τ=r+re is not exactly solvable for **p** and *r*.

It is well known that biased TOA equations are often described by τ=r+re with *r* representing the clock bias between the transmitter and receiver. Interestingly, in $\tau =r+\bar{r}e$, $\bar{r}$ is specified as the average of the difference between {*τ*_*i*_} and {*r*_*i*_}. As implied in the proof of Theorem 2, $\bar{r}$ is actually the least squares solution of *r* to τ=r+re.

## Theoretical verification

Some primary results on matrix irreducibility are needed for proving Theorem 1.

**Lemma 1** *(Corollary 6.2.27 [16]) An irreducibly diagonally dominant matrix is non-singular.*

**Lemma 2** *(Proposition 1.1 [17]) If* **A**_1_ and **A**_2_ are irreducible, **A**_12_ ≠ 0 and **A**_21_ ≠ 0, *then* $\left[\begin{array}{cc} A_{1} & A_{12}\\ A_{21} & A_{2} \end{array}\right]$ *is irreducible*.

### Proof of Theorem 1

Direct calculations give

$$\begin{array}{c} \bar{E}=D_{\bar{e}}-{\bar{E}}_{e}-{\bar{E}}_{e}^{\prime }, \end{array}$$ (13)

where ${\bar{E}}_{e}=\left[e_{ij}^{2}\right]$, and $D_{\bar{e}}$ is the diagonal matrix formed by $\bar{e}=\left[{\bar{e}}_{i}\right]$ with

$$\begin{array}{c} {\bar{e}}_{i}=\sum_{j\neq i}(e_{ij}^{2}+e_{ji}^{2}),\;i=1,2,\cdots ,m. \end{array}$$ (14)

Obviously, $\bar{E}$ is symmetric, and diagonally dominant, which is a). It is also at least positive semi-definite which is part of b) due to

$$\begin{array}{c} {\left|E\circ (xe^{\prime }-ex^{\prime })\right|}^{2}={\left|E\circ T_{x}\right|}^{2}=x^{\prime }\bar{E}x\geq 0 \end{array}$$ (15)

following from some simple properties of Hadamard products [16], such as

$$\begin{array}{c} A\circ B=B\circ A,\;(A\circ B)\circ C=A\circ B\circ C, \end{array}$$ (16)

and identities

$$\begin{array}{ccc} E\circ T_{x}=D_{x}E-ED_{x},\;\text{tr}(D_{x}AD_{y}B^{\prime }) & = & x^{\prime }(A\circ B)y \end{array}$$ (17)

for arbitrary vectors **x** and **y**, and arbitrary matrices **A**, **B** and **C**, all with compatible dimensions. This verifies c) due to the existence of decomposition $\bar{E}={\left({\bar{E}}^{\frac{1}{2}}\right)}^{\prime }{\bar{E}}^{\frac{1}{2}}$. It also leads to b) because of rank deficiency of $\bar{E}$ in view of $\bar{E}e=0$ from (13). Moreover, ${\bar{\tau }}^{\prime }e=0$ follows from the skew symmetry of $E\circ E\circ T-{\left(E\circ E\circ T\right)}^{\prime }$, which implies $\text{rank}[\;\bar{E},\;\bar{\tau }\;]<m$. A deductive verification of irreducibility of $\bar{E}$ and $\text{rank}\;\bar{E}=m-1$ in the following completes the proof of d) and e).

Denote **E** by **E**_*m*_ and $\bar{E}$ by ${\bar{E}}_{m}$, and set $E_{1}={\bar{E}}_{1}=0$. For *m* = *k* > 0,

$$\begin{array}{c} E_{k+1}=[\begin{array}{cc} E_{k} & e_{c,k+1}\\ e_{r,k+1}^{\prime } & 0 \end{array}] \end{array}$$ (18)

with

$$\begin{array}{ccc} e_{c,k+1} & = & {\left[\begin{array}{ccc} e_{1,k+1} & \cdots & e_{k,k+1} \end{array}\right]}^{\prime }, \end{array}$$ (19)

$$\begin{array}{ccc} e_{r,k+1} & = & {\left[\begin{array}{ccc} e_{k+1,1} & \cdots & e_{k+1,k} \end{array}\right]}^{\prime }, \end{array}$$ (20)

and

$$\begin{array}{c} {\bar{E}}_{k+1}=[\begin{array}{cc} {\bar{E}}_{k}+D_{{\bar{e}}_{k+1}} & -{\bar{e}}_{k+1}\\ -{\bar{e}}_{k+1}^{\prime } & {\bar{e}}_{k+1} \end{array}], \end{array}$$ (21)

where ${\bar{e}}_{k+1}=\left[e_{i,k+1}^{2}+e_{k+1,i}^{2}\right]$ for *i* = 1, …, *k*, and ${\bar{e}}_{k+1}$ is given in (14) for *i* = *m* = *k* + 1. Clearly, ${\bar{E}}_{2}$ is irreducible and $\text{rank}\;{\bar{E}}_{2}=1$ due to ${\bar{e}}_{2}={\bar{e}}_{2}=e_{12}^{2}+e_{21}^{2}\neq 0$ in view of (4).

Suppose ${\bar{E}}_{k}$ is irreducible and $\text{rank}\;{\bar{E}}_{k}=k-1$ for *k* > 2. Trivially, irreducibility of ${\bar{E}}_{k}$ implies the same of ${\bar{E}}_{k}+D_{{\bar{e}}_{k+1}}$. In view of (14), ${\bar{e}}_{k+1}\neq 0$ and ${\bar{e}}_{k+1}\neq 0$, which ensures irreducibility of ${\bar{E}}_{k+1}$ according to Lemma 2. Moreover, ${\bar{E}}_{k}+D_{{\bar{e}}_{k+1}}$ is irreducibly diagonally dominant, and hence non-singular according to Lemma 1. This verifies $\text{rank}\;{\bar{E}}_{k+1}\geq k$, where the inequality cannot hold nevertheless because ${\bar{E}}_{k+1}e=0$ follows from (13).

### Proof of Theorem 2

By definition, the left side of (9) specifies least squares solutions to (3). Using (6), (16), (17), **D**_**e**_ = **I**, and some simple properties of the trace of matrix products, the following is obtained

$$\begin{array}{ccc} & & {\left|E\circ (T\;-\;R)\right|}^{2}\\ & \;\;=\;\; & {\left|E\circ T\right|}^{2}\;-\;2\text{tr}({\left(E\circ T\right)}^{\prime }\left(D_{r}E-ED_{r}\right))+\\ & & \text{tr}(D_{r}\left(E^{\prime }E+EE^{\prime }\right)D_{r}\;-\;E^{\prime }D_{r}ED_{r}\;-\;D_{r}E^{\prime }D_{r}E)\\ & \;\;=\;\; & {\left|E\circ T\right|}^{2}\;-\;2r^{\prime }\bar{\tau }\;+\;r^{\prime }\bar{E}r\\ & \;\;=\;\; & {\left|E\circ T\right|}^{2}\;-\;{\bar{\tau }}^{\prime }{\bar{E}}^{+}\bar{\tau }\;+\;{\left|{\bar{E}}^{\frac{1}{2}}r\;-\;{\bar{E}}^{\frac{1}{2}}{\bar{E}}^{+}\bar{\tau }\right|}^{2} \end{array}$$ (22)

$$\begin{array}{ccc} & \;\;=\;\; & {\left|E\circ T\right|}^{2}\;-\;\tau^{\prime }\bar{E}\tau \;+\;{\left|{\bar{E}}^{\frac{1}{2}}\left(\tau -r\right)\right|}^{2}. \end{array}$$ (23)

Completing the square in (22) has used $\bar{E}={\left({\bar{E}}^{\frac{1}{2}}\right)}^{\prime }{\bar{E}}^{\frac{1}{2}}$ and $\bar{\tau }=\bar{E}{\bar{E}}^{+}\bar{\tau }$ due to c) and e) of Theorem 1 respectively. The second term in (22) and that in (23) are obtained from $|{\bar{E}}^{\frac{1}{2}}{\bar{E}}^{+}\bar{\tau }{|}^{2}={\bar{\tau }}^{\prime }{\bar{E}}^{+}\bar{\tau }$, symmetry of $\bar{E}$ (and hence ${\bar{E}}^{+}$), and ${\bar{E}}^{+}\bar{E}{\bar{E}}^{+}={\bar{E}}^{+}$. Since the first two terms in (23) are independent of **p**, the equivalence in (9) is then proved.

Direct calculations produce

$$\begin{array}{ccc} {\left|T_{\tau }\;-\;R\right|}^{2} & = & {\left|\left(\tau \;-\;r\right)e^{\prime }\;-\;e{\left(\tau \;-\;r\right)}^{\prime }\right|}^{2}\\ & = & 2m{\left(\tau -r\right)}^{\prime }\left(\tau -r\right)-2{\left(\tau -r\right)}^{\prime }e{\left(\tau -r\right)}^{\prime }e\\ & = & 2m(|\tau -r{|}^{2}-m{\bar{r}}^{2})\\ & = & 2m{\left|\tau -r-\bar{r}e\right|}^{2} \end{array}$$ (24)

which verifies the equivalence in (10).

From $\bar{E}e=0$ (and hence $e^{\prime }\bar{E}e=0$) and $\bar{E}={\left({\bar{E}}^{\frac{1}{2}}\right)}^{\prime }{\bar{E}}^{\frac{1}{2}}$, ${\bar{E}}^{\frac{1}{2}}e=0$ follows. Consequently, ${\bar{E}}^{\frac{1}{2}}\left(\tau -r\right)={\bar{E}}^{\frac{1}{2}}\left(\tau -r-re\right)$ for arbitrary **p** and *r*. By setting $r=\bar{r}$ and noticing **Ab**| ≤ |**A**||**b**| for arbitrary **A** and **b**, the first inequality in (11) is verified. Setting partial differentiation ${\partial |\tau -r-re|}^{2}/\partial r=0$ leads to $r=\bar{r}$, which implies $|\tau -r-re|\geq |\tau -r-\bar{r}e|$ for arbitrary **p** and *r*, and hence confirms the second inequality. From this, the third inequality follows immediately.

### Proof of Corollary 1

Noting **R** = **T**_**r**_ and from (23) with **T**_**x**_ replacing **R**, it is ready to have

$$\begin{array}{c} \text{arg}\;\underset{x}{\text{min}}|E\circ (T-T_{x}){|}^{2}=\text{arg}\;\underset{x}{\text{min}}{\left|{\bar{E}}^{\frac{1}{2}}\left(\tau -x\right)\right|}^{2}. \end{array}$$

The general least squares solution of **x** to ${\bar{E}}^{\frac{1}{2}}\left(\tau -x\right)=0$ is

$$x={\left({\bar{E}}^{\frac{1}{2}}\right)}^{+}{\bar{E}}^{\frac{1}{2}}\tau +y,$$

where **y** is arbitrary but subject to ${\bar{E}}^{\frac{1}{2}}y=0$. Considering singular value decomposition ${\bar{E}}^{\frac{1}{2}}=U\Sigma^{\frac{1}{2}}V^{\prime }$ and hence $\bar{E}=U\Sigma V^{\prime }$, it is ready to verify

$$\begin{array}{c} {\left({\bar{E}}^{\frac{1}{2}}\right)}^{+}{\bar{E}}^{\frac{1}{2}}\tau ={\left({\bar{E}}^{\frac{1}{2}}\right)}^{+}{\bar{E}}^{\frac{1}{2}}{\bar{E}}^{+}\bar{\tau }={\bar{E}}^{+}\bar{\tau }=\tau . \end{array}$$

Recalling ${\bar{E}}^{\frac{1}{2}}e=0$ and rank ${\bar{E}}^{\frac{1}{2}}=m-1$, **y** must be parallel to **E**, which leads to the general expression y=-re with *r* being an arbitrary scalar.

## Illustrative example

A numerical example of 3D positioning is used to illustrate the developed results. When used for illustrating effects of different sets of range and range-difference equations on positioning, simulated datasets are considered most effective than practical datasets. This is because noise levels for range-difference measurements and inaccuracy of the reference locations could be easily set and examined in numerical examples. It is however not the case in a real setup where inaccuracies of the measurements and reference locations are coupled with the uncertainty of the object location.

Let four references be located precisely at $\left[\;{\bar{p}}_{1},\;{\bar{p}}_{2},\;{\bar{p}}_{3},\;{\bar{p}}_{4}\right]=\left[\;0,\;I\;\right]$, but inexactly known as $p_{i}={\bar{p}}_{i}+n_{i}$ with noise vector $n_{i}∼N\left(0,\sigma_{p}^{2}I\right)$. Measurements of range differences are produced according to *τ*_*ij*_ = *r*_*ij*_ + *n*_*ij*_ in (1) with *r*_*ij*_ = *r*_*i*_−*r*_*j*_ and $r_{k}=\left|p-{\bar{p}}_{k}\right|$ for *k* = *i*, *j* and noise variable $n_{ij}∼N\left(0,\sigma_{n}^{2}\right)$. An object is with its coordinates randomly generated within range (1, 10) as

$$\begin{array}{c} p={\left[\begin{array}{ccc} 9.8115 & 6.0293 & 1.1923 \end{array}\right]}^{\prime }. \end{array}$$ (25)

The standard deviations are set as *σ*_*p*_ = 0.1% and *σ*_*n*_ = max{|*r*_*ij*_|} × 1%. For instance, one simulation run produced reference and measurement matrices as

$$\begin{array}{c} {}[p_{1}\;p_{2}\;p_{3}\;p_{4}]\;=\;[\;\;\begin{array}{cccc} -0.0006\; & 1.0002\; & 0.0010\; & -0.0009\\ 0.0015\; & -0.0005\; & 1.0003\; & 0.0002\\ 0.0027\; & -0.0014\; & 0.0012\; & 0.9993 \end{array}\;\;],\\ T\;=\;[\begin{array}{cccc} 0 & 0.8177 & 0.4903 & 0.0568\\ -0.8502 & 0 & -0.3522 & -0.7858\\ -0.5027 & 0.3437 & 0 & -0.4278\\ -0.0676 & 0.7765 & 0.4345 & 0 \end{array}].\; \end{array}$$

As expected, since the noise levels are low, deviations of {**p**_*i*_} from $\left\{{\bar{p}}_{i}\right\}$ are insignificant, and also due to *r*_*ij*_ = −*r*_*ji*_, **T** is approximately skew symmetric.

To find the object position, the unconstrained multivariable minimization algorithm fminsearch in MATLAB has been used. In numerical minimization, referring to Theorem 2, the squared forms of the criteria

$$\begin{array}{c} \begin{array}{ccc} \text{a)}\;\;|E\;\circ \;(\;T\;-\;R\;)|;\; & \text{b)}\;\;|{\bar{E}}^{\frac{1}{2}}\;\left(\;\tau \;-\;r\;\right)|; & \text{c)}\;\;|\;T_{\tau }\;-\;R\;|;\\ \text{d)}\;\;|\tau \;-\;r\;-\;\bar{r}e|; & \text{e)}\;\;|\tau \;-\;r\;-\;re| & \text{f)}\;\;|\tau \;-\;r| \end{array} \end{array}$$ (26)

were used, and the initial estimate of (**p**, *r*) was taken as $\left(p/2,\bar{r}/2\right)$ in each simulation run.

**Fig 1:**
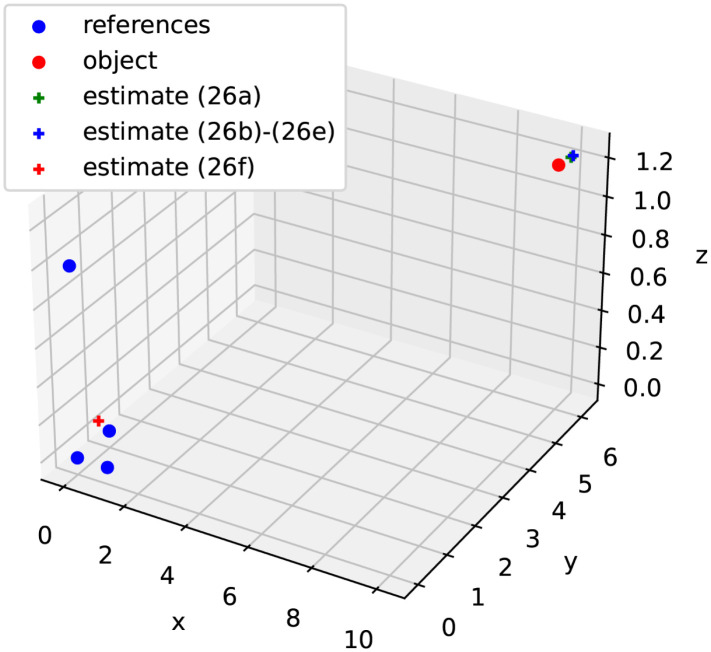
Geometric setup for positioning a 3D object with four references.

Fig 1 indicates the geometric setup for positioning a 3D object with four references. Table 1 shows the estimates of the object position in 50 runs of simulations under the noise conditions stated above for the reference locations and rage-difference measurements. The estimates using the squared criteria (26a) and (26b) are very close to each other, while those using criteria (26b) to (26e) are indistinguishable from each other. Due to *r* = − 11.81 on average over the 50 simulation runs, the estimates using the squared criteria (26f) are too poor to be useful. This value of *r* should not be interpreted as a clock bias because the generation of the ranges and their noisy measurements had not introduced an offset in each run of the simulations. In fact, even if introduced, a bias in TOA measurements cannot be recovered from TDOA measurements.

The best and worst estimates are determined with respect to $|p-\hat{p}|$ which cannot be evaluated in real applications nevertheless. Worst cases of the randomly generated reference locations and range difference measurements ought to be responsible for worst estimates of **p** in Table 1. This is because the evaluations of criteria (26b) to (26e) have generated insignificant values at level 10^−7^ which corresponds to level 10^−14^ produced by least squares. As expected, if no noise is added to the reference locations and range-difference measurements, all estimates, except for those using (26f), recover **p** up to a computational error at the level of 10^−14^ close to the machine epsilon 10^−16^.

**Table 1:** Estimation of the object position using algorithm fminsearch with initial estimate $\left(p/2,\bar{r}/2\right)$ and use of noise corrupted references and measurements of all range differences.

	position estimation $\hat{p}\;$	$|p-\hat{p}|$	criterion & value
best	[9.7939, 5.9975, 1.2346]	0.06	(26a) 1.06 × 10^−2^
mean	[10.0746, 6.1680, 1.2261]	1.93	(26a) 1.88 × 10^−2^
worst	[16.1242, 9.7248, 1.8867]	7.35	(26a) 2.33 × 10^−2^
best	[9.8219, 6.0204, 1.2209]	0.03	(26b) 5.33 × 10^−7^
mean	[10.1188, 6.1966, 1.2329]	1.21	(26b) 6.66 × 10^−7^
worst	[13.7830, 8.3322, 1.5230]	4.60	(26b) 7.45 × 10^−7^
best	as above	as above	(26c) 0.97 × 10^−7^
mean			(26c) 2.59 × 10^−7^
worst			(26c) 1.18 × 10^−7^
best	as above	as above	(26d) 0.48 × 10^−7^
mean			(26d) 1.27 × 10^−7^
worst			(26d) 0.59 × 10^−7^
best	as above	as above	(26e) 1.34 × 10^−7^
mean			(26e) 1.39 × 10^−7^
worst			(26e) 0.56 × 10^−7^
best	[0.4381, 0.2926, 0.1767]	11.04	(26f) 1.68
mean	[0.4362, 0.2926, 0.1772]	11.04	(26f) 1.68
worst	[0.4345, 0.2928, 0.1781]	11.04	(26f) 1.68

It is interesting to know how availability of measurements affects estimations. Consider the following three cases of availability of range-difference measurements in **T** = [*τ*_*ij*_], where no weighting is applied to available measurements:

Case 1: [*τ*_*ij*_] for all *i* and *j* ≠ *i* (12 measurements);
Case 2: [*τ*_*ij*_] for all *i* and *j* > *i* (6 measurements);
Case 3: *τ*_12_, *τ*_14_, *τ*_23_ and *τ*_24_ (4 measurements).

In Table 2, three averaged values of each minimization criterion and estimation error correspond to the above three cases. On average, use of more measurements is shown to have better estimations. This indicates, as expected, that all available range-difference measurements should be used for positioning. Use of different squared criteria in (26), except for (26f), in minimization has produced estimations of the object position with similar or identical averaged errors of $|p-\hat{p}|$ in all case of measurement availability. This shows the desired close performances of minimizing the biased range Eq (8) and minimizing the original range-difference Eq (3) by least squares. The poor performance with (26f) indicates unsuitability of the range equation τ=r for positioning.

**Table 2:** Evaluations of least squares criteria and estimation errors over 50 simulation runs with averaged values in a bracket corresponding to cases 1, 2 and 3 of measurement availability.

evaluation of criteria in (26)	estimation error $|p-\hat{p}|$
(26a) = (1.88, 1.16, 0.68) × 10^−2^,	(1.53, 2.52, 4.91)
(26b) = (6.66, 4.96, 3.15) × 10^−7^,	(1.21, 2.17, 3.58)
(26c) = (2.59, 2.70, 2.49) × 10^−7^,	(1.21, 2.17, 3.58)
(26d) = (1.30, 1.35, 1.25) × 10^−7^,	(1.21, 2.17, 3.58)
(26e) = (1.39, 1.54, 1.56) × 10^−7^,	(1.21, 2.17, 3.58)
(26f) = (1.68, 1.68, 1.68),	(11.04, 11.04, 11.04)

## Concluding remarks

Given *m* references, following the procedure in [18] on the basis of processing individually received signals, up to *m*(*m* − 1)/2 TDOA measurements could be made available. The procedure in [19] on the basis of processing each pair of received signals could produce up to *m*(*m* − 1) TDOA measurements. The current work has proposed a general method for use of these multiple TDOA measurements for positioning.

While weighted least squares positioning has been considered in this work, it has not addressed issues of selection of weighting coefficients. As widely used, for instance in [5, 6] among others, an obvious choice of weightings is the inverse variances of the measurement noise components, which could be obtained from a processor of generating TDOA measurements such as those in [18, 19].

Minimizing the difference between the noise-corrupted TDOA measurement matrix and the well-structured matrix formed by a range-measurement vector is the denoising problem explored in [4]. The current work directly addresses the problem of minimizing TDOA equations with respect to the object location and automatically obtains the range red-measurement vector. The current focus is on positioning in a general setting with a simultaneous treatment of missing and weighted TDOA measurements. The current work has however not considered the issue of eliminating outlier measurements as examined in [4] and comprehensively explored in [20].

A numerical example has been used to illustrate the theoretical results presented in this paper. To evaluate effectiveness of the proposed method in real applications, an experiment could be designed. It would need a high-precision positioning system for referencing, and apply the method to a low-precision TDOA dataset. A practical application of the results in this paper could be in wireless sensor networks [21] with massive low-cost miniature sensors often randomly deployed in a geographical area, where a sensor could be localized by using a huge number of TDOA measurements from references including already localized sensor nodes [22].
